# Consistency of Phenolic Compounds in Plant Residues Parts: A Review of Primary Sources, Key Compounds, and Extraction Trends

**DOI:** 10.1021/acs.jafc.5c01868

**Published:** 2025-04-29

**Authors:** Monique Martins Strieder, Isadora Lopes de Oliveira, Felipe Sanchez Bragagnolo, Vitor Lacerda Sanches, Rodrigo Stein Pizani, Leonardo Mendes de Souza Mesquita, Mauricio Ariel Rostagno

**Affiliations:** † Multidisciplinary Laboratory of Food and Health (LabMAS), School of Applied Sciences (FCA), Universidade Estadual de Campinas, Rua Pedro Zaccaria 1300, 13484-350 Limeira, São Paulo Brazil

**Keywords:** phenolic compounds networking, fruit waste, selective extraction, inline detections

## Abstract

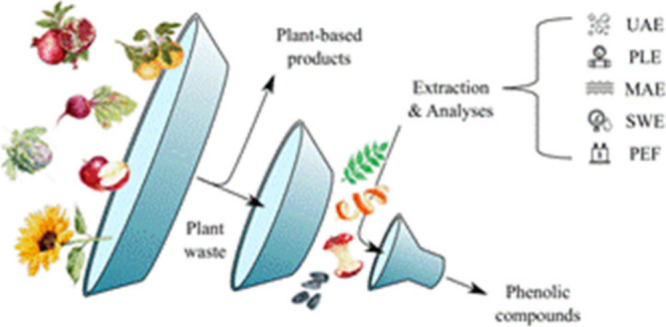

A significant challenge in valorizing food waste is the accurate extraction and identification of metabolites, as the composition of phenolic compounds varies by plant species, part, growth conditions, and processing. This review examined phenolic compounds in plant residue groups (leaves/stalks, peels/husks, pulp/pomace, and seeds) to verify the predominance of specific compounds in the same plant groups, establishing a comprehensive database. This database may be helpful for future studies that seek sources of a given compound or develop solvents to extract phenolic compounds from a specific material. Moreover, the primary plant residues and trends in extracting and analyzing these compounds were reviewed. The predominance of specific compounds within these groups, such as luteolin in plant leaves and stalks, was observed. Most studies focus on extracts with the highest total phenolic content (TPC), limiting insights into how extraction variables affect the target compounds. Chromatographic methods vary according to sample type, column, and conditions, shifting toward reducing acetone/methanol use, shortening the analysis time, and integrating inline UV–vis detection. This perspective highlights plant residue parts rich in specific phenolics, contributing to more targeted, selective, and sustainable extraction methodologies.

## Introduction

Food loss and waste are global concerns, considering their environmental, social, and economic impacts. In this context, target 12.3 of the Sustainable Development Goals (SDGs) aims to reduce global food waste throughout production and supply chains by 2030. Fruits and vegetables, among the food sectors, contributed the most to waste generation.^1^ These materials include citrus and coffee peels, grape and apple pulp/seeds from juice, coffee, wine, and cider production, olive pomace after oil extraction, and leaves and stalks rendered unusable after fruit harvesting. Despite retaining valuable compounds for food chains, these materials contribute to soil contamination from landfill saturation and air pollution through methane emissions as waste.^2^ The issues are exacerbated by the high final cost of the product, considering the expenses associated with waste disposal, leading to environmental, economic, and social challenges.

One way to mitigate waste generation is by using solid residues to obtain high-value ingredients, which are still persistent in those biomasses. For example, dietary fibers and bioactive compounds, such as phenolic compounds, carotenoids, terpenes, terpenoids, and alkaloids, have been acquired from plant waste.^3,4^ These compounds can be used in several industrial domains, from food/feed ingredients and even in pharmaceutical formulations.^5,6^ Phenolic compounds stand out among these ingredients because they are present in most plant tissues and have several biological activities, mainly but not limited to antioxidant, antimicrobial, anti-inflammatory, and antiproliferative activities.^7^

The phenolic compound profile in plant waste can vary significantly based on species, plant part, growth conditions, and processing methods.^8^ This behavior becomes particularly complex when studying phenolic compounds due to their numerous derivatives and their wide variety of compound types (flavonoids, phenolic acids, stilbenes, and lignans). Moreover, these bioactive compounds have been extracted from different plant matrices employing a wide range of solid–liquid extraction techniques aiming at efficiency while following the principles of green chemistry based on social, environmental, and economic issues. Hence, an essential and desired component in this realm is an analytical tool (mainly based on chromatographic analysis associated with UV–vis and mass detectors) for identifying and quantifying the obtained compounds and guiding pertinent applications.^9^ However, biologically, it is anticipated that certain plant species may share similar phenolic compositions in specific parts due to their protective roles. Therefore, this review sought to analyze experimental studies to determine whether the same phenolic compounds are consistently extracted from the corresponding plant residue parts, including leaves/stalks, peels/husks, pulp/pomace, and seeds. Moreover, extraction and chromatographic techniques to isolate and analyze these compounds were reviewed, emphasizing trends in sustainable development.

## Main Phenolic Compounds in Plant Residues Groups

**Figure 1:**
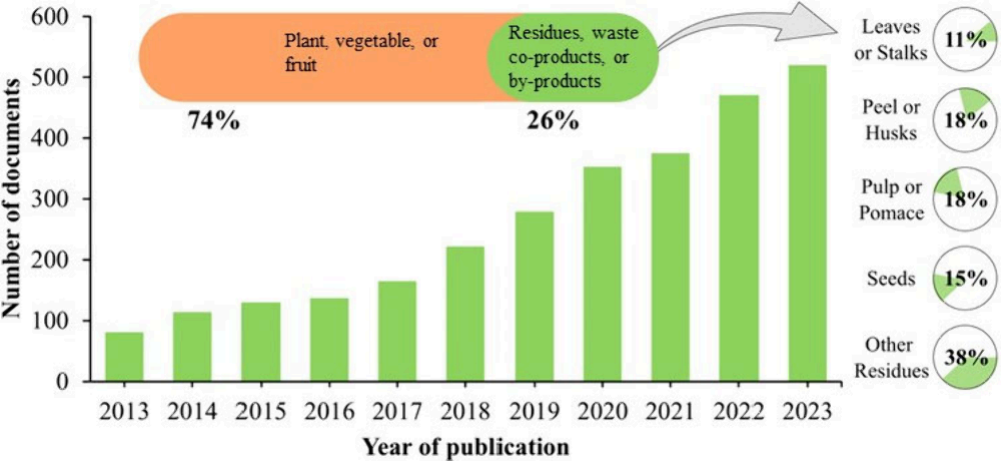
Distribution of the number of documents between 2013 and 2023 using the keywords in the Scopus database: “phenolic AND compounds AND extraction AND residues OR coproducts OR waste OR byproduct”, percentage of publications using the same keywords (green) and percentage using “phenolic AND compounds AND extraction AND plant OR vegetable OR fruit” (orange), and pie chart demonstrating the distribution of searches using the keywords “phenolic AND compounds AND extraction AND residues OR coproducts OR waste OR byproduct AND chromatography AND food leaves” or stalks/peel or husks/pulp or pomace/seeds.

A search in the Scopus database for studies on the extraction of phenolic compounds from plant materials identified 7322 documents published between 2013 and 2023. In contrast, when focusing specifically on extraction from plant residues, only 2847 documents were found. This result indicates the need to explore food waste further to obtain phenolic compounds for a more comprehensive use of natural resources. Figure 1 presents the results acquired in the Scopus database: (i) the distribution of the number of documents between 2013 and 2023 about phenolic compounds extracted from residues, waste, coproducts, or byproducts, (ii) the percentage of publications about the same theme (in green) and percentage about phenolic compounds extracted from plant, vegetable, or fruit (in orange), and (iii) pie charts demonstrating the distribution of searches using food leaves or stalks/peel or husks/pulp or pomace/seeds.

Although fewer studies evaluate the extraction of phenolic compounds from residues, coproducts, waste, and byproducts (26%) than from plant, vegetable, and fruit (74%), this number of studies has been growing from 2013 to 2023. Spain, followed by Brazil, Italy, China, and Portugal, is the country that does the most research on this theme, according to the Scopus search from 2013 to 2023. This more intense activity of Spain and Brazil on the topic may be related to the production of plant products. Spain is the leading producer of fruits and vegetables in Europe, and Brazil is the third-largest producer of fruits globally.^10^ Peel/husks and pulp/pomace are the solid plant residues most investigated as sources of phenolic compounds, followed by seeds and food leaves/stalks, according to this Scopus search.

**Figure 2:**
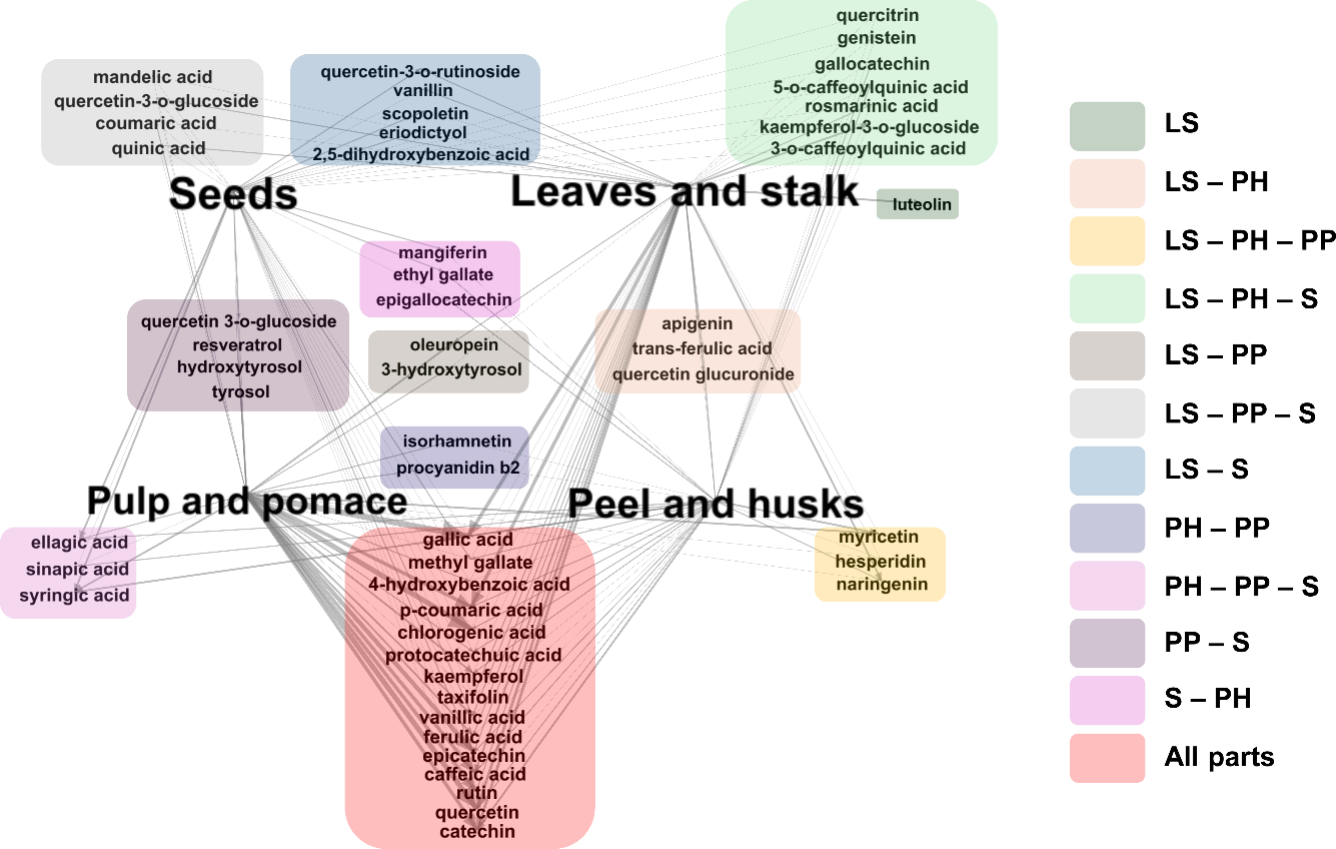
Network analysis of the phenolic compound distribution across different plant parts: Leaves and Stems (LS), Peels and Husk (PH), Pulp and Pomace (PP), and Seeds (S).

Aiming to verify the phenolic compounds most present in each group of residues, a search in Scopus was performed by adding the “chromatography” keyword (phenolic AND compounds AND extraction AND residues OR coproducts OR waste OR byproducts AND chromatography AND leaves OR stalk/peel OR husks/pulp OR pomace/seeds), founding fewer documents: leaves and stalk (80), peel and husks (105), pulp and pomace (85), and seeds (73). Each document was analyzed, and the identified phenolic compounds were extracted into an Excel table (Supporting Information (SI)). Thus, compounds within different parts of plants were cataloged in an Excel data set detailing the number of occurrences of each compound (SI, Table S1). Only compounds cited more than three times were selected as nodes to ensure a focus on the most prevalent compounds to prepare the data for network analysis. Edges were defined based on the presence of these compounds in specific plant parts, with weights assigned according to the documented occurrences. The software Gephi 0.10.1, an open-source network analysis tool, was employed to construct a bipartite network consisting of nodes representing compounds and plant parts with edges denoting the presence of these compounds in the parts. Nodes were visually differentiated by type and significance by using color and size. Layout algorithms such as ForceAtlas2 were employed to distribute nodes to minimize overlap and to maximize readability spatially. Additionally, the thickness of each edge in the network was proportional to the number of occurrences of the corresponding compound in the assigned plant part, visually emphasizing the most prevalent connections. The networking is presented in Figure 2.

As shown in Figure 2, individual phenolic compounds were more likely to be identified and quantified exclusively in specific plant parts, such as luteolin in leaves and stalk, apigenin, *trans*-ferulic acid, and quercetin glucuronide in leaves, stalk, peels, and husks, oleuropein and 3-hydroxytyrosol in leaves, stalk, pulp, and pomace, and ellagic, sinapic, and syringic acids in seeds, peels, and husk. Otherwise, some phenolic compounds have been found in all plant parts and can be more related to the plant species, such as gallic acid, methyl gallate, *p*-coumaric acid, chlorogenic acid, protocatechuic acid, kaempferol, taxifolin, vanillic acid, ferulic acid, epicatechin, caffeic acid, rutin, quercetin, and catechin.

The high citation frequency of these compounds may reflect their importance in plant metabolism and their value in various applications, from health-related benefits in the nutraceutical and pharmaceutical industries to their use as natural preservatives in the food industry. Furthermore, the differential distribution of these compounds, as depicted in Figure 2, underscores the need for targeted extraction methods and the potential for optimizing the use of specific plant parts in industrial applications.

## Main Sources of Phenolic Compounds in Plant Residues Groups

Leaves are laminar, green plant organs that are primarily responsible for performing photosynthesis. On the other hand, stems function as conduits for plant sap, supporting this plant’s overall structure. Despite their significance during plant growth and their rich composition in bioactive compounds, these materials are considered solid residues once they are detached from the plants. Consequently, numerous studies have explored the potential utilization of this biomass as sources of phenolic compounds. Olive leaves have received substantial attention as valuable sources of phenolic compounds, as evidenced by three studies in Table 1 and the 13 studies found by Scopus from 2013 to 2023 (Supporting Information). This heightened interest can be attributed to their notably high TPC compared to those of other plant species (Table 1). In contrast, stems have received comparatively less attention as a source of phenolic compounds in scientific research, accounting for 12 studies of total found for leaves and stalks (Supporting Information).

**Table 1:** Extraction and Analysis of Phenolic Compounds Obtained from Plant Leaves and Stems^a^

reference	plant solid residue	best extraction conditions	extraction solvent	phenolic compounds analysis	TPC/TFC	main phenolic compounds
Pande et al. (2017)^46^	Indian bamboo (*Bambusa tulda*) leaves	Soxhlet	methanol–water (4:1, v/v)	HPLC-ESI-QTOF-MS Supelco C18 column (10 cm × 2.1 mm, 2.7 μm), 25 °C, water (0.1% formic acid) and ACN, 43 min	221 mg GAE/g/135 mg QE/g	caffeic acid, coumaroylquinic acid, dihydroxybenzoic acid, 5-feruloylquinic acid, *p*-coumaric acid, orientin, sinapic acid, ferulic acid, vitexin, quercitrin, homoorientin, isovitexin, and tricin
		t: 6 h				
		SM: 10 g				

Santos et al. (2016)^47^	Brazilian cassava (*Manihot esculenta* Crantz) leaves	heat-reflux	methanol–water (50:50, v/v)	HPLC, Ascentis C18 column (25 cm × 4.6 mm, 5 μm), 15 °C, 280 nm, 2% acetic acid in water and methanol/water/acetic acid (70:28:2, v/v/v), 50 min	n-d	gallic acid, gallocatechin, catechin, epigallocatechin, and chlorogenic acid
		T: 80 °C				
		t: 45 min				
		50 mL/g				

Henriques et al. (2017)^48^	Portuguese kiwifruit (*Actinidia deliciosa*) pruning leaves	infusion	boiling water	HPLC-DAD, 100 RP8 column (5 μm), 200–500 nm, 0.05% acid in water and methanol, 25 min	n-d	quinic acid, proanthocyanidin B, proanthocyanidin C, quercetin-3-*O*-rutinoside-7-*O*-glucoside, quercetin-3-*O*-rhamnoside-7-*O*-glucoside, myricitrin, rutin, and quercitrin
		t: 10 min				
		10 mL/g				

Yu et al. (2017)^49^	Chinese stevia (*Stevia rebaudiana*) stems	maceration overnight and boiling	distilled water	HPLC-PDA-ESI-MS, TKSgel ODS column (30 cm × 21.5 mm, 5 μm), 250 nm, 0.1% acid in ACN and ACN–water 60:40 (0.1% acid), 60 min	46.14 mg GAE/g	vanillic acid, protocatechuic acid, caffeic acid, chlorogenic acid, and crypto chlorogenic acid
		t: 30 min				
		10 mL/g				

Cvetković et al. (2018)^50^	aronia (*Aronia melanocarpa*) leaves	maceration	ethanol–water (50:50, v/v)	UHPLC-MS, C18 column (5 cm × 2.1 mm, 1.9 μm), 25 °C, 0.1% acid in water and methanol, 15 min	20 mg GAE/g	chlorogenic acid, quercetin-3-*O*-galctoside, quercetin-3-*O*-rutinoside, and quercetin-3-*O*-glucoside
		t: 90 min				
		5 mL/g				

Da Silva et al. (2018)^51^	Brazilian cinnamon tree (*Nectandra grandiflora* Nees Lauraceae) leaves	Soxhlet	ethanol–water (96:4, v/v)	UPLC-PDA-ESI-MS Acquity, C18 column (10 cm × 2.1 mm, 1.7 μm), 40 °C, 0.1% acid in water and methanol, 30 min	279 mg GAE/g/151 mg QE/g	myricetrin-rhamnoside, quercetin-rhamnoside, and kaempferol-rhamnoside
		t: 24 h				
		20 mL/g				

Radojković et al. (2018)^52^	Serbian mulberry (*Morus nigra*) leaves	MAE	ethanol–water (1:1, v/v)	HPLC-PDA, Phenomenex Gemini C18 column (25 cm × 4.6 mm, 5 μm), 25 °C, 320 and 360 nm, 0.1% acid in methanol and water, 90 min	19.7 mg GAE/g	caffeic acid, chlorogenic acid, cinnamic acid, naringin, and rutin
		T: 120 °C				
		t: 28 min				
		Po: 1500 W				
		48.3 mL/g				

Battistella Lasta et al. (2019)^53^	Brazilian beetroot (*Beta vulgaris* L.) leaves and stems	PLE	ethanol (99.8%)	LC-ESI-MS/MS, Synergy column (15 cm × 2.0 mm, 4 μm), 30 °C, ethanol/water (95.5% v/v) and 0.1% acid in water, 23 min	252 mg GAE/g	ferulic acid, vitexin, iso-quercetin, quercetin, and sinapldehyde
		T: 40 °C				
		P: 10 MPa				
		3 mL/min				

Chihoub et al. (2019)^54^	Tunisian turnip (*Brassica rapa* L.) leaves	infusion	boiling distilled water	UHPLC-DAD-MS, ODS-2C18 column (15 cm × 4.6 mm, 3 μm), 35 °C, 280 to 370 nm, 1% acid in water and ACN, 10 min	n-d	caffeic acid, ferulic acid, quercetin-3-*O*-glucoside, kaempferol-3-*O*-glucoside, and isorhamnetin-*O*-pentoside
		100 mL/g				
		t: 5 min				

Wang et al. (2019)^55^	Chinese raspberry (*Rubus idaeus* L.) leaves	UAE	diethyl ether and ethyl acetate with 2 M HCl after neutralized with NaOH	HPLC-DAD-ESI-TOF-MS, Zorbax Eclipse Plus C18 column (25 cm × 4.6 mm, 5 μm), 30 °C, 200 to 600 nm, 0.1% acid in water and ACN, 50 min	31.7 mg GAE/g/35.1 mg RE/g	gallic acid, chlorogenic acid, epicatechin, ellagic acid, procianidins trimer isomer, kaempferol derivatives, quercetin derivatives, and rutin
		T: 85 °C				
		Po: 320 W				
		t: 30 min				
		15 mL/g				

Acquadro et al. (2020)^56^	Italian grapevine (*Vitis vinifera* L.) pruning leaves	UAE	methanol–water (70:30, v/v)	HPLC-PDA-MS/MS RP-amide column (10 cm × 2.1 mm, 2.7 μm), 30 °C, 220–450 nm water/acid and ACN/acid (999:1, v/v), 60 min	n-d	caftaric acid, rutin, hyperoside, quercetin-3-*O*-glucoside, kaempferol-3-*O*-glucuronide, kaempferol-3-*O*-rutinoside, resveratrol, and isorhamnetin
		T: 30 °C				
		t: 15 min				
		F: 40 kHz				
		100 mL/g				

Hou et al. (2020)^57^	Chinese red sage (*Salvia miltiorrhiza*) Bunge leaves	UAE	methanol–water (80:20, v/v)	HPLC-DAD, YMC-Pack ODS-A column (25 cm × 4.6 mm, 5 μm), 25 °C, 280 nm, 0.1% acid in water and ACN, 80 min	70.58 mg GAE/g	caffeic acid, rutin, isoquercetin, rosmarinic acid, and salvianolic acid B
		T: 55 °C				
		t: 45 min				
		Po: 200 W				
		F: 40 kHz				
		20 mL/g				

Pollini et al. (2020)^58^	Italian goji berry (*Lycium barbarum*) leaves	UAE	methanol	UPLC-ESI-QTOF-MS, Agilent 120 EC-C18 column (10 cm × 3 mm, 2.7 μm), 0.1% acid in water and ACN, 15 min	7.75 mg GAE/g	syringic acid, chlorogenic acid, salicylic acid, caffeic acid, vanillic acid, *p*-coumaric acid, sinapic acid, and vanillin
		T: 45 °C				
		t: 30 min				
		P: 180 W				
		60.6 mL/g				

Esposito et al. (2021)^59^	Italian red and white grape (*Vitis vinifera*) leaves	MAE	water	HPLC-UV, C18 Luna column (25 cm × 3 mm, 5 μm), 280 and 360 nm, 0.5% acid in water and methanol, 67 min	104.38 μg QE/mg	gallic acid, vanillic acid, quercetin derivatives, kaempferol derivatives
		t: 2 min				
		Po: 300 W				
		10 mL/g				

Ben-Othman et al. (2021)^60^	Estonian apple tree (*Malus domestica* Borkh.) leaves	UAE	ethanol–water (70:30, v/v)	UPLC-MS C18 PFP column (10 cm × 2.1 mm), 40 °C, 1% acid in water and in methanol, 43 min	57.74 mg GAE/g	chlorogenic acid, *p*-coumaric acid, caffeic acid, phloridzin, phloretin, quercetin-3-glucoside, quercetin-3-galactoside, quercetin-3-rhamnoside, rutin, and kaempferol-3-glucoside
		T: 25 °C				
		t: 14.4 min				
		F: 20 kHz				
		Po: 400 W				
		10 mL/g				

López-Salas, et al. (2021)^28^	artichoke (*Cynara scolymus* L.) bracts and stems	PLE	ethanol	HPLC-DAD-ESI-TOF-MS, Agilent Zorbax Eclipse Plus C18 column (15 cm × 4.6 mm, 1.8 μm), 0.1% acid in water and methanol, 45 min	n-d	quinic acid, chlorogenic acid, rosmarinic acid, cynarin isomers, luteolin derivatives, and apigenin derivatives
		T: 120 °C				
		t: 20 min				
		P: 1500 psi				
		4 g				

Sánchez-Gutiérrez et al. (2021)^61^	Spanish olive (*Olea europaea* L.) leaves	Soxhlet	ethanol–water (50:50, v/v)	HPLC-DAD, Kinetex EVO C18 100A column (25 cm × 4.6 mm, 5 μm), 254 °C, 280, and 340 nm, 0.1% acid in water and ACN, 55 min	76.1 mg GAE/g	hydroxytyrosol, verbascoside, luteolin derivatives, apigenin derivatives, and oleuropein
		t: 5 h				
		8 mL/g				

Jara et al. (2022)^62^	Guayule (*Parthenium argentatum* A. Gray) tree leaves	stirring	methanol–water (80:20, v/v)	UHPLC-ESI-MS ACE Excel C18-PFP column (10 cm × 2.1 mm, 2 μm), ACN, 9 min	29.86 g GAE/kg	gluconic acid, quinic acid, ferulic acid, citric acid, gallic acid, vanillic acid, caffeic acid, hydroxybenzoic acid derivatives, and caffeoylquinic acid derivatives
		T: RT				
		t: 24 h				
		50 mL/g				

Maravić et al. (2022)^22^	Serbian sugar beet (*Beta vulgaris* L.) leaves	MAE	ethanol–water (70:30, v/v)	HPLC-DAD Zorbax Eclipse XDB-C18 column (5 cm × 4.6 mm, 1.8 μm), 30 °C, 280 nm, 0.1% acid in methanol and in water, 30 min	17.17 mg GAE/g	catechin, vitexin, isovitexin, flavone derivatives, *p*-coumaric acid, and sinapic acid
		t: 10 min				
		Po: 600 W				
		10 mL/g				

Márquez et al. (2022)^63^	olive mill “Arbequina” (*Olea europaea*) leaves	stirring	ethanol–water (80:20, v/v)	HPLC-DAD Spherisorb ODS2 C18 column (25 cm × 4.6 mm, 5 μm), 280 nm, 0.1% acid in water and ACN–water (70:30, v/v), 50 min	52 mg GAE/g	quinica acid, caffeic acid, sologanoside, rutin, ligstroside, hydroxytyrosol, luteolin, derivatives, and oleuropein derivatives
		T: 40 °C				
		t: 30 s				
		10 mL/g				

Míguez et al. (2022)^64^	Spanish fortune herb (*Tradescantia fluminensis*) leaves and stems	stirring	distilled water	HPLC-ESI-MS/MS Phenomenex Luna C18 column (15 cm × 2 mm, 3 μm), 0.1% acid in water and in ACN, 17 min	4.21 mg GAE/g	*p*-coumaric acid, ferulic acid, sinapic acid, protocatechuic acid, salicylic acid, syringic acid, naringenin, and quercetin
		T: 25 °C				
		t: 6 h				
		100 mL/g				

Souza et al. (2022)^65^	Brazilian pitanga cherry (*Eugenia uniflora* L.) leaves	MAE	choline chloride and lactic acid (1:3) with 20% water	HPLC-DAD, waters phenyl-hexyl column (15 cm × 4.6 mm, 5 μm), 35 °C, 270 nm, 1% acid in water and ethanol–water (95:5), 30 min	n-d	gallic acid, ellagic acid, and quercetin
		T: 39 °C				
		t: 52 min				
		Po: 800 W				
		20 g/g				

Siamandoura and Tzia (2023)^29^	olive (*Olea europaea* L. *var. argentata*) leaf	HAE	ethanol–water 70%, v/v)	HPLC-DAD, Hypersil C18 column (25 cm × 4.6 mm, 5 μm), 280 nm, methanol and 2% acid in methanol, 45 min	5.12 mg GAE/g	oleuropein, hydrohytyrosol, and rutin
		T: 60 °C				
		2000 rpm				

De Montijo-Prieto et al. (2023)^35^	fermented avocado (*Persea americana* Mill., Lauraceae) leaves	UAE	ethanol–water 80:20, v/v	HPLC-ESI-TOF-MS Shield RP18 column (10 cm × 2.1 mm, 1.7 μm), 30 °C, 1% acid in water and ACN, 25 min	n-d	chlorogenic acid, ferulic acid, quercetin, catechin, and rutin
		2×				
		t: 15 min				
		30 mL/g				

a UAE: ultrasound-assisted extraction, MAE: microwave-assisted extraction, PLE: pressurized liquid extraction, HAE: homogenate-assisted extraction, T: temperature, RT: room temperature, t: extraction time, SM: sample mass, P: pressure, Po: Power, A: amplitude, F: frequency, n-d: nondetermined, ACN: acetonitrile; TPC: total phenolic content; TFC: total flavonoid content; GAE: gallic acid equivalent; QE: quercetin equivalent; (UH)PLC: (ultrahigh) performance liquid chromatography; DAD/PDA: photodiode-array detector; ESI: electrospray ionization; (Q)TOF: quadrupole time-of-flight; MS: mass spectrometry.

Plant peels and husks constitute the outer protective, structuring, organizational, and space-limiting layers of vegetables and fruits, shielding them from external environmental factors. Typically, these parts of plant materials are discarded during the processing and by end consumers.^11^ Peel flours acquired from plant materials have been incorporated into the formulations of bakery items, jams, and meat-based products, extending their shelf life, improving their oxidative stability, and enhancing their nutritional value.^12^ These beneficial effects are primarily attributed to bioactive compounds, predominantly phenolic compounds. Consequently, numerous studies have developed processes using peel and husk residues as sources of phenolic compounds. Citrus (13), mango (11), pomegranate (7), and avocado (6) peel and husk residues are the most studied sources of phenolic compounds of the peel/husk group (Supporting Information). The citrus occurrence is probably due to its widespread global consumption and its high generation of solid waste.^13^

Pulp or pomace is the main edible part of plant organs and is rich in carbohydrates, fibers, vitamins, and other minor compounds. This part of the plant disperses the seeds, enabling future dissemination of the species. Typically, these pulps serve as primary food sources for consumers. However, in the food industry, particularly within juice, olive oil, and wine production, a partial portion of the constituents within these materials is extracted, resulting in pulp or pomace as a coproduct. These residues may represent a good amount of the plant; for example, *Rosa roxburghii* Tratt pomace accounts for nearly 50% of the whole flower.^14^ Pulp wastes most studied as sources of phenolic compounds are grapes (21), olives (19), and apples (4) (Supporting Information).

Seeds are the mature and already fertilized ovules of plants, formed by tegument or bark, embryo, and endosperm, responsible for plants’ reproduction and dispersal. Generally, they are discarded as waste when processing plant-based products, such as juices.^15^ However, these plant materials often possess a higher concentration and variety of phenolic compounds, particularly in flavonoids. Flavonoids, in turn, play a significant role in plant survival through their involvement in crucial metabolic pathways.^16−18^ Grape seeds have been the most studied seed source of gallic acid and other phenolic compounds (Supporting Information). However, the potential of seeds as a source of phenolic compounds has been little explored. Few studies that evaluate seeds as sources of phenolic compounds identify the extracted compounds. For example, Restrepo-Serna et al. (2022)^19^ conducted a cost analysis study on the recovery of bioactive compounds, followed by ethanol production and electricity from avocado seeds. The authors suggest an increase in the profitability of the process, highlighting the economic potential of a biorefinery focused on obtaining bioactive compounds from the seeds. However, the authors did not quantify extracted phenolic compounds nor did they identify them.

## Extraction Techniques and Conditions

Among the extraction techniques, the studies demonstrated that over the years, conventional methods such as Soxhlet, heat-reflux, infusion, magnetic stirring, and maceration have been replaced by those with intensified solid–liquid extraction performances, mainly based on using other energy sources such as ultrasound-assisted extraction (UAE), microwave-assisted extraction (MAE), pulsed electric field extraction (PEF), pressurized liquid extraction (PLE), and subcritical water extraction (SWE). Hybrid extraction techniques combining UAE and MAE or PLE and solid-phase extraction (SPE) also appear promising for increasing extractive processes’ efficiency and selectivity. This phenomenon can be attributed to the limitations of conventional techniques, which typically necessitate extended extraction times and the utilization of non-GRAS (Generally Recognized as Safe) organic solvents to attain satisfactory extraction yields. Tables 1, 2, 3, and 4 provide an overview of some selected studies that employed different extraction techniques and chromatographic methods to obtain and analyze phenolic compounds from food leaves/stems, peel/husks, pulp/pomace, and seeds, respectively. Furthermore, the total phenolic content (TPC) determined by the Folin–Ciocalteu spectrophotometer method was assessed.

**Table 2:** Extraction and Analysis of Phenolic Compounds Obtained from Plant Peels and Husks^a^

reference	source	best extraction conditions	extraction solvent	chromatographic analysis	TPC/TFC	phenolic compounds
Nayak et al. (2015)^66^	*Citrus sinensis* peel	MAE	water–acetone (51:49, v/v)	HPLC-DAD, C18 column (4.6 mm × 250 mm, 5 μm), 30 °C, 254, 289, 520, 300, and 700 nm, 6:94 (v/v) acetic acid in water, 100% ACN, 40 min	12.20 mg GAE/g	chlorogenic acid, catechin, rutin, gallic acid, *p*- coumaric acid, caffeic acid, and ferulic acid
		T: 80 °C				
		t: 122 s				
		Po: 500W				
		F: 2.45 kHz				
		25 mL/g				

Wang et al. (2018)^67^	red orange peel	UAE	water–ethanol (85:15, v/v)	HPLC-DAD-ESI-M2, reverse- phase amide column (15 cm × 4.6 mm), 25 °C, 320 nm, water in ACN, 40 min	n-d	tangeretin and nobiletin
		T: 50 °C				
		t: 40 min				
		Po: 150 W				
		F: 20 kHz				
		20 mL/g				

Kaderides et al. (2019)^68^	pomegranate peel	MAE	water–ethanol (50:50, v/v)	HPLC UV–vis, reverse-phase column (250 mm × 4.6 mm, 5 μm), 30 °C, 254 and 280 nm, 5% acetic acid in water, and CAN, 12 min	199.4 mg GAE/g	phenolics acids, flavonoids, catechins, and procyanidins
		T: 30 °C				
		t: 4 min				
		Po: 600W				
		F: 2.45 kHz				
		60 mL/g				

Mei et al. (2020)^69^	*Xanthoceras sorbifolia* husks	UAE	water–ethanol (60:30, v/v)	HPLC UV–vis, ODS-C18 column (250 mm × 4.6 mm, 5 μm), 25 °C, 0.1% formic acid and ACN, 20 min	23.16 mg GAE/g	gallic acid, protocatechuic acid, epicatechin, rutin, rutinoside, and quercetin
		T: 40 °C				
		t: 40 min				
		F: 100 kHz				
		100 mL/g				

Figueroa et al. (2021)^39^	avocado peel	MAE	water–ethanol (36:64, v/v)	HPLC-ESI-TOF/GTOF-MS, C18 column (150 nm × 4.6 nm, 1.8 μm), 25 °C, formic acid in water and ACN, 55 min	72.04 mg GAE/g	flavonoids, catechins, procyanidins, and phenolic acids
		T: 130 °C				
		t: 39 min				
		Po: 850 W				
		30 mL/g				

Dewi et al. (2022)^70^	cacao pod husk	MAE	water–ethanol (50:50, v/v)	HPLC-VWD, reverse-phase column (250 mm × 4.6 mm, 5 μm), 30 °C, 30 °C, 280 nm, 0.1% of orthophosphoric acid in water, and ACN, 50 min	100.4 mg GAE/g	catechin, quercetin, epicatechin, gallic acid, coumaric acid, and protocatechuic acid
		T: 50 °C				
		t: 5 min				
		Po: 120 W				
		F: 2.45 kHz				
		80 mL				

Estrada-Gil et al. (2022)^24^	rambutan peel	UAE + MAE	water (1:16, m/v)	HPLC-MS, reverse-phase (150 mm × 2.1 mm, 3 μm), 30 °C, 245, 280, 320, 550 nm, formic acid and ACN, 60 min	176.38 mg GAE/g	geraniin, corilagin, and ellagic acid
		UAE				
		t: 20 min				
		F: 25 kHz				
		MAE				
		T: 70 °C				
		t: 5 min				
		F: 2450 kHz				

Anticona et al. (2022)^71^	citrus peel	UAE	water–ethanol (50:50, v/v)	HPLC-UV, C18 column (250 mm × 4.6 mm, 5 μm), 280 nm, formic acid in water and ACN, 80 min	n-d	catechin, caffeic acid, vanillic acid, quercetin, naringenin, apigenin, and hesperidin
		T: 35–40 °C				
		t: 30 min				
		Po: 400 W				
		F: 20 kHz				
		45 mL				

Wang et al. (2023)^38^	brocade orange peels	UAE	water– methanol– DMSO (1:4:5)	UHPLC-Q-TOF-MS, C18 column (4.6 mm × 100 mm, 3.5 μm), 35 °C, formic acid and methanol, and HPLC-DAD, Aq-C18 column, same gradient, 35 °C	24.97 mg GAE/g	caffeic acid, sinapic acid, ferulic acid, narigin, hesperidin, luteolin, rutin, sinensetin, nobiletin, and tangeretin
		T: 26 °C				
		t: 30 min				
		Po: 60 W				
		F: 40 kHz				
		17.6 mL/g				

Gómez-Urios et al. (2022)^72^	orange peel	magnetic stirring	choline chloride and glycerol ratio of 1:2 with 25% water	HPLC UV–vis, C18 column (250 nm × 4.6 nm, 5 μm), 280 nm, 5% acid formic in water and 40% in ACN, 80 min	903 mg GAE/g	ascorbic acid, flavonoids, and polyphenols
		T: 45 °C				
		t: 20 min				
		10g/mL				

Maimulyanti et al. (2023)^34^	coffee husk from west Java, Indonesia	magnetic stirring	choline chloride and proline ratio of 1:1with 50% water	HPLC-PDA, reverse phase column (250 mm × 4.6 mm, 5 μm), 30 °C, 327 nm, 50 mM acid in distillate water and 50 mM acid in ACN, 10 min	10.07 mg GAE/g	chlorogenic acid
		T: 80 °C				
		t: 30 min				
		Po: 200 W				
		F: 34 kHz				
		10 mL/g				

Strieder et al. (2024)^44^	Arabic coffee husk: green coffee beans 60:40 (w/w)	PLE	water	HPLC-PDA, Kinetex C18 column (100 mm × 4.6 mm, 2.6 μm), 50 °C, 270 and 325 nm, 0.1% acetic acid in water and in ACN, 9 min	n-d	chlorogenic acid and caffeine
		T: 125 °C				
		P: 150 MPa				
		flow: 2 mL/min				
		50 mL/g				

a UAE: ultrasound-assisted extraction; MAE: microwave-assisted extraction; PLE: pressurized liquid extraction; HAE: homogenate-assisted extraction; TPC: total phenolic content; TFC: total flavonoid content; (UH)PLC: (ultrahigh) performance liquid chromatography; DAD/PDA: photodiode-array detector; ESI-M2: electrospray ionization coupled mass spectrometry; (Q)TOF: quadrupole time-of-flight; MS: mass spectrometry, VWD; variable wavelength detector; T: temperature; RT: room temperature; t: extraction time; SM: sample mass; P: pressure; Po: Power; A: amplitude; F: frequency; TPC: total phenolic content; TFC: total flavonoid content; GAE: gallic acid equivalent; n-d: nondetermined; ACN: acetonitrile.

Based on the data presented in Tables 1–4, it is evident that there is no clear differentiation in extraction methods targeting specific compounds from different plant groups. Instead, optimal conditions are more closely associated with each plant species. For example, extracting anthocyanins from pomace/pulp using acidified water or deep eutectic solvents (DES) enhances pigment stabilization and extraction (Table 3: Lončarić et al. (2020)^20^ and de Souza Mesquita et al. (2023)^21^). Additionally, it is worth noting that most studies do not compare the developed method to other extraction techniques or alternative technologies. Furthermore, they typically analyze the phenolic compound profile only in the extract with the highest TPC by using chromatographic techniques. In this sense, the considerations below will be generally mentioned, not focusing on the plant residue groups.

**Table 3:** Extraction, Separation, and Analysis of Phenolic Compounds Obtained from Pulp and Pomace^a^

reference	source	extraction technique	extraction solvent	chromatographic analysis	TPC/TF (best results)	phenolic compounds
Ribeiro et al. (2015)^73^	grape pomace (*Vitis vinifera* and *Vitis labrusca*)	HAE	ethanol–water (40:60, v/v)	HPLC-DAD-MS/MS C18 column (50 m × 4.6 m i.d., 5 μm), room temperature, 520 nm, water–formic acid–ACN (95:2:3, v/v/v) and water–formic acid–ACN (48:2:50, v/v/v), 45 min	41.24 mg GAE/g	anthocyanins, gallic acid, vanillic acid, syringic acid, *trans*-cinnamic acid, caffeic acid, chlorogenic acid, *p*-coumaric acid, catechin, quercetin, rutin, kaempferol, and trans-resveratrol
		t: 24 h				
		T: 25 °C				
		1:50 (m/v)				

Viganó et al. (2016)^74^	passion fruit bagasse	PLE	ethanol–water (50:50, v/v) and 75:25, v/v)	UHPLC-MS/MS, C18 column (3.0 mm i.d., 100 mm, 2.6 μm), 40 °C, 0.1% formic acid in water: methanol, 20 min	55.2 mg GAE/g	piceatannol, scirpusin B
		T: 70 °C				
		P: 10 MPa				

Kheirkhah et al. (2019)^40^	Hayward kiwifruit pomace	SWE	water	HPLC-DAD, Luna C18 column (250 mm × 4.6 mm, 5 μm), 35 °C, 280, 320, and 360 nm, 0.1% formic acid in water and methanol, 40 min	60.53 mg CaE/g	(+)-catechin, chlorogenic acid, *p*-coumaric acid, protocatechuic acid, and caffeic acid
		T: 200 °C				
		t: 90 min				
		P: 50 bar				
		100 mL/g				

Lončarić et al. (2020)^20^	blueberry pomace	PEF	water–ethanol–HCL (49:50:1, v/v)	HPLC-DAD/LC-(HESI)-MS/MS, C18 Kinetex column (150 mm × 4.5 mm, 2.6 μm), 50 °C, 190–600 nm, 1% formic acid in water and in methanol, 22 min	10.52 mg/GAE	catechin, epicatechin procyanidin B1, and procyanidin B2
		pulses: 100				
		E: 20 kV/cm				
		W_T_: 41 kJ/kg				
		50 mL/g				

Heravi et al. (2022)^75^	grape (*Vitis vinifera* L.) pomace	maceration	water, ethanol–water (50:50, v/v), and ethanol	HPLC-UV, C18 column, 25 °C, 280 nm, 0.02% acidified water and methanol, 50 min	205.33 mg GAE/g	hydroxybenzoic acids, catechins, procyanidins, caffeoylquinic acids
		v: 500 rpm				
		T: 80 °C				
		8 mL/g				

.Carpentieri et al (2022)^25^	white grace pomace	PEF	ethanol–water (50:50, v/v)	HPLC-PDA, C18 reverse phase column (4.6 mm × 250 mm, 5 μm), 280, 310, 360 nm, water/methanol (0.1% phosphoric acid, v/v), 35 min	4.07 mg GAE/g	*p*-coumaric acid, quercetin, epicatechin
		t: 23 min				
		T: 50 °C				
		E: 3.8 kV/cm				
		W_T_: 10 kJ/kg				

Frum et al. (2022)^76^	red fermented pomaces (*Vitis vinifera* L.)	UAE bath	methanol– water– hydrochloric acid (70:29:1, v/v/v)	HPLC-DAD, C18 column (250 mm × 4.6 mm, 5 μm), 25 °C, 280 nm, acidified water and methanol (96:4 v/v), 70 min	6.60 mg GAE/g	chlorogenic acid, rutin, ferulic acid, catechin, gallic acid, cinnamic acid, resveratrol, syringic acid, quercetin, and caffeic acid
		t: 30 min				
		T: 40 °C				
		0.05 mL/g				

Huang et al. (2022)^14^	defatted *Rosa roxburghii* Tratt pomace	HAE (3×)	ethanol–water (70:30, v/v)	UHPLC-ESI-QTOF-MS/MS C18 column (2.1 mm × 75 mm, 2 μm), 35 °C, 0.1% formic acid in water and ACN, 30 min	224.92 mg GAE/g	gallic acid, gallocatechin, epigallocatechin, catechin, hydroxybenzoic acid, epicatechin, ellagic acid, ferulic acid, and quercetin
		T: RT				
		t: 2 h				
		15 mL/g				

Garcia-Montalvo et al. (2022)^77^	grape and apple pomace	HAE	ethanol–water (70:30, v/v)	HPLC-DAD, C18 column (250 mm × 4.6 mm, 0.5 μm), 25 °C, 1.5% formic acid in water and ACN, 80 min	68.46 mg GAE/g	flavonols, cinnamic acids, and anthocyanins
		v: 500 rpm				
		T: 90 °C				
		40 mL/g				

Belghith, et al. (2022)	olive pomace	HAE	rthanol–water (60:40, v/v)	UPLC-DAD, C18 column (2.1 mm × 50 mm, 1.7 μm), 1% formic acid in water and CAN, 14 min	4.76 mg tyrosol equiv/g	3-hydroxytyrosol, tyrosol, caffeic acid, and *p*-coumaric acid
		T: 50 °C				

Danielski et al. (2022)^78^	red guava (*Psidium guajava* L.) pomace	UAE	ethanol–water (30:70, v/v)	HPLC ESI-MS/MS, Synergi column (2.0 mm × 150 mm, 4.0 μm), 30 °C, 374 nm, 0.1% formic acid in water and methanol, 17 min	23.48 mg GAE/g	ellagic acid, vanillic acid, gallic acid, and isoquercetin
		Po = 800 W				
		T = 25 °C				
		t: 1 h				
		BUAE				
		F: 40 kHz				

Mesquita et al. (2022)^27^	acerola pomace	SWE	water	UPLC-ESI-QTOF-MS, BEH UPLC column (150 mm × 2.1 mm, 1.7 μm), 40 °C 0.1% of formic acid in water and ACN, 19.1 min	348.3 mg GAE/g	kaempferol, quercetin, and isorhamnetin
		P: 10 MPa				
		T: 110 °C				
		4 mL/min				
		t: 15 min				

Da Silva et al. (2023)^79^	apple pomace	PLE-SPE	water and ethanol	HPLC-PDA, C18 (100 mm × 4.6 mm, 2.6 μm), 48 °C, 260, 280, and 350 nm, 0.1% acidified water and ACN with acetic acid, 10 min	n-d	furfurals, chlorogenic acids, flavonoids and PLD
		T: 80 °C				
		P: 10 ± 0.5 MPa				
		flow: 1 mL/min				

de Souza Mesquita et al. (2023)^21^	Brazilian berry waste (*Plinia cauliflora*)	PLE-SPE	eutectic mixture with chloride choline and lactic acid	HPLC-PDA, C18 column (100 mm × 4.6 mm, 2.6 μm), 50 °C, 520 nm, acidified water (0.25 mol L^–1^ citric acid) and ethanol, 2 min	n-d	anthocyanins
		T: 40 °C				
		P: 1500 psi				
		flow: 1.5 mL/min				
		30 mL/g				

a UAE: ultrasound-assisted extraction; BUAE: Bath-type ultrasound-assisted extraction; MAE: microwave-assisted extraction; PLE: pressurized liquid extraction; PEF: Pulsed electric field extraction; HAE: homogenate-assisted extraction; SWE: subcritical water extraction; T: temperature; RT: room temperature; t: extraction time; P: pressure; Po: Power; A: amplitude; F: frequency; E: PEF field strength; W_T_: PEF energy input; n-d: nondetermined; TPC: total phenolic content; TFC: total flavonoid content; GAE: gallic acid equivalent; HPLC: high-performance liquid chromatography; DAD: diode array detector; ACN: acetonitrile.

Maravić et al. (2022)^22^ showed that MAE produced the highest extraction yield compared to conventional solid–liquid extraction, UAE, PLE, and SWE from sugar beet leaves. MAE operates by generating electromagnetic waves that result in cellular disruption within the plant material. This disruption occurs due to the heat generated, which induces dipole rotation in organic molecules and subsequently disrupts hydrogen bonding. Consequently, this mechanism enables enhanced mass transfer between the raw material and the solvent, as Akhtar et al. (2019)^23^ described. Hybrid extraction techniques combining UAE and MAE also appear promising for increasing extractive processes’ efficiency. Estrada-Gil et al. (2022)^24^ observed the highest TPC extraction yield (176.38 mg GAE/g of dry rambutan peel) from Mexican rambutan (*Nephelium lappaceum* L.) peel by combining UAE and MAE. The MAE methodology (2450 MHz for 5 min until the system reached 70 °C) or UAE (25 kHz for 20 min until the system reached 70 °C) alone provided less than half the extraction yield that provided the hybrid technique (UAE + MAE). In this case, the coupling of emerging technologies probably facilitated the extraction, adding different mechanisms to the extractive system and increasing mass transfer.

Carpentieri et al. (2022)^25^ observed that PEF increased the extraction yield of TPC (+8%) and flavonoid content (+31%) of extracts obtained from white grape pomace, decreasing the extraction time (by 23–103 min) and solvent consumption (by 3–12%) concerning a solid–liquid extraction performed in the same conditions (50 °C for 23 min). The PEF pulses probably affected the permeability of grape pomace membranes, enhancing the release of intracellular compounds, favoring the solvent’s penetration and the extraction of compounds in a shorter time.^26^ Mesquita et al. (2022)^27^ studied phenolic compound extraction from acerola by SWE, obtaining higher extraction yields in a short time of 15 min than the classic 6 h of Soxhlet extraction using ethanol.

Maravić et al. (2022)^22^ acquired higher polyphenol yields from sugar beet leaves employing SCW by increasing the temperature from 100 to 150 °C. This result was associated with changes in the water dielectric constant that decrease by increasing the temperature at high pressure (20 MPa). The water dielectric constant at high pressures and temperatures is closer to that of organic solvents, such as methanol (ε = 33). Thus, the high-pressure water probably favored the extraction of less polar compounds from the sugar beet leaves. López-Salas et al. (2021)^28^ presented the different dielectric constants of water, ethanol, and hydroethanolic mixtures at various temperatures under high pressure (40–200 °C). They observed lower dielectric constants for the solvents by increasing their temperatures at high pressures. Ethanol at 120 °C exhibited the lowest dielectric constant (ε = 19), resulting in the highest total phenolic content (TPC) from *C. scolymus* L. The highest temperature (200 °C) also produced a high yield of compounds. However, at 200 °C, most phenolic compounds can undergo some thermal alteration or degradation. These changes cannot be verified through a TPC spectrometer analysis. Therefore, in this case, it would be interesting to evaluate the extracts using more informative techniques, such as the chromatographic techniques associated with UV–vis or mass detectors, to identify and quantify the compounds more accurately.

Siamandoura and Tzia (2023)^29^ observed the highest extraction yield using the conventional homogenate-assisted extraction method compared to MAE, UAE, and high hydrostatic pressure extraction from olive leaves. Despite the leaves presenting fewer rigid tissues, favoring the simple solid–liquid extraction compared to other plant parts and explaining the better results acquired by the conventional homogenate-assisted extraction method, the authors employed different extraction conditions for each technique, making direct comparisons challenging. For instance, homogenate-assisted extraction was evaluated at various temperatures (40 or 60 °C) and homogenization speeds (4000 or 12,000 rpm) for 30 min. Conversely, in high-pressure-assisted extraction, which yielded the lowest extraction yield, they examined different pressure levels (300 and 600 MPa) and extraction times (5 and 10 min) at a constant temperature of 25 °C. As heat enhances mass transfer, comparing a technique performed at room temperature directly with a heat-assisted technique would be undue.

In this context, it is essential to acknowledge that apart from the chosen extraction technique, the specific conditions employed during the extraction process also significantly influence the results obtained, considering the efficiency and sustainability of the process. Among extraction conditions, solvent, temperature, and time are those that most affect extractions and therefore the most studied.

## Solvent

Over the years, as can be seen in Tables 1–4, efficient organic solvents for extracting phenolic compounds such as methanol, diethyl ether, and ethyl acetate have been giving space to ethanol, water, hydroethanolic mixtures, and DES. The exchange of solvents is generally accompanied by emerging technologies that intensify extraction through energies other than heat (ultrasound, pressure, microwaves, and others) to obtain higher extraction yields. This trend comes from sustainability requirements and green chemistry that seek to use fewer toxic solvents that could cause damage to natural resources due to their disposal.

Aiming at the extraction of target compounds, software such as COSMO-RS and employing Hansen solubility parameters (HSP) have been used as valuable tools for the development of new solvents as well as for understanding solvent–solute interactions.^21,30,31^ These tools assist in the selection of solvents and the development of new ones, seeking greener options that can be directly applied to pharmaceutical and food products. They have been widely used for developing DES in particular. A DES is a mixture of two or more compounds forming a single-phase system with a melting point lower than those of its individual components, exhibiting significant negative deviations from ideal behavior. These solvents are formed by the complexation of a hydrogen bond acceptor (HBA) with a hydrogen bond donor (HBD), creating strong hydrogen bond interactions and nonvolatile solvents with high viscosity.^32^ Furthermore, they are usually formulated with natural components that provide sustainability to the extraction process due to their low toxicity, efficiency, and possibility of ready-to-use applications.^33^

The most used DES combine the hydrogen bond acceptor (HBA) choline chloride with various hydrogen bond donors (HBDs), such as glycerol, glucose, citric acid, or proline (Maimulyanti et al. (2023).^34^ Additionally, water is often incorporated to reduce viscosity and enhance performance in the extraction processes. However, the application of these solvents is still in its early stages and requires careful evaluation, particularly considering the final applications where solvent components remain in the extract. This visualization is crucial because the separation of DES from the extract is complex and typically involves adsorption steps.

## Temperature

The temperature is one of the most critical factors in the extraction processes; for example, increasing UAE temperature positively impacted the extraction of phenolic compounds from sugar beet leaves.^22^ Improved mass transfer is achieved through a higher rupture of the plant cell wall, enhancing the release and recovery of bioactive compounds from the interior cell content at higher temperatures (from 30 to 70 °C). Despite its importance, some studies do not report the temperature used to carry out UAE extractions, which may hinder their procedure reproduction. De Montijo-Prieto et al. (2023)^35^ employed a fixed UAE condition to obtain phenolic compounds from avocado leaves. However, they did not report the temperature achieved during or after the extraction. The acoustic cavitation produced by applying ultrasound energy in a liquid medium generated heat, increasing the temperature that may favor the mass transfer.^36^ In this sense, measuring and reporting the extraction temperature profile is necessary.

The optimal extraction temperature to obtain phenolic compounds from plant peel and husks was observed from 26 to 130 °C, depending on the technique, employed conditions, and target compounds.^37,38^ Wang et al. (2023),^38^ increasing the UAE temperature from 20 to 26 °C increased the TPC and TFC content of extracts acquired from brocade orange peel. However, raising the temperature from 26 to 50 °C reduced the TPC and TFC values. In other words, temperature elevation promotes mass transfer by significantly affecting the solvent solubility. However, beyond a certain threshold, thermal degradation of the extracted compounds. Since the analysis focused on TPC, which specific compounds may have undergone degradation remains unclear. On the other hand, Figueroa et al. (2021)^39^ acquired the highest TPC yield from avocado peel by increasing MAE temperature from 40 to 130 °C. They explained that higher temperatures decrease solvent viscosity, enhancing its mobility and solubility and consequently improving the extraction efficiency of the target compounds. They also noted that numerous studies have reported the degradation of thermosensitive phenolic compounds at temperatures exceeding 130 °C. However, since they did not evaluate the temperature’s effect on compounds within the extract, it remains uncertain whether any compounds are thermally degraded.

Kheirkhah et al. (2019)^40^ employed the highest temperature (200 °C) to obtain phenolic compounds from a kiwifruit pomace using SWE. Under subcritical water conditions, the dielectric constant of water decreases with rising temperature. Consequently, thermal vibrations take place among the molecules, causing a weakening of the hydrogen bonds. This weakening reduces surface tension, enabling water to swiftly infiltrate the matrix, facilitating more efficient extraction processes.^2^ Kheirkhah et al. (2019)^40^ evaluated the subcritical water temperature (175, 200, and 225 °C) observing that 225 °C allowed higher yields of TPC and TFC. However, increasing the temperature from 175 to 225 °C and the extraction time from 10 to 180 min also favored the Maillard reaction during SCW, increasing the brown color of the extracts and accumulating melanoidins as the reaction time increased. Despite this, the authors highlighted that melanoidins have antioxidant potential similar to that of phenolic compounds.

Most studies presented in Table 4 for extracting phenolic compounds from seeds did not optimize or do not report the extraction temperature. Okur et al. (2021)^41^ evaluated PLE and UAE of phenolic compounds from spent coffee at the fixed temperature of 25 °C. Dorneles and Noreña (2020)^42^ did not report the extraction temperature employed to obtain phenolic compounds from *Araucaria angustifolia* bracts using MAE. On the other hand, Guzmán-Lorite et al. (2022)^43^ studied pomegranate seeds as a primary source of proteins rather than phenolic compounds. Thus, the optimized temperature of 170 °C was chosen based on the protein extraction yield. Therefore, considering possible thermal degradation, a lower temperature would have been more suitable for extracting phenolic compounds in this last study. In this context, future studies should assess the impact of temperature not only on TPC but also on the profile of extracted phenolic compounds. These results would provide a deeper understanding of how the temperature influences the preservation or degradation of these compounds.

**Table 4:** Extraction, Separation, and Analysis of Phenolic Compounds Obtained from Seeds^a^

reference	source	extraction technique	extraction solvent	chromatographic analysis	TPC/TF (best results)	main phenolic compounds
Oliveira et al. (2014)^80^	cherry seeds	PLE	ethanol	HPLC/ESI/MS, LiChrospher C18 (150 mm × 4.6 mm, 5 μm), 25 °C, 0.1% formic acid in water and methanol, 50 min	1.60 mg GAE/g	ellagic acid, kaempferol, and quercetin derivate
		T: 70 °C				
		t: 8 min				
		P: n-d				
		7 mL/g				

Deng et al. (2016)^81^	kiwi seeds	HAE	acetone 60%	HPLC-ECD, Zorbax C18 (150 mm × 4.6 mm, 5 μm) proceeded by a C18 guard column (20 mm × 4.0 mm, 5 μm), methanol and water, 35 min	53.73 mg GAE/g	protocatechuic, *p*-hydroxybenzoic, caffeic, *p*-coumaric, and ferulic acids
		T: 40 °C				
		t: 80 min				
		12 mL/g				

Dorta et al. (2014)^82^	mango seeds	MAE	acetone 50%	HPLC-ESI-Q-TOF-MS with DAD, C18 reversed-phase column (250 mm × 4.6 mm, 5 μm), 30 °C, 0,1% formic acid in water and ACN, 280 and 360 nm, 75 min	44.76 g/100 g	maclurin-3-C-β-d-glucoside and mangiferin
		T: 30 °C				
		t: 60 min				
		Po: 500 W				
		50 mL/g				

Wang et al. (2020)^83^	*Camellia sinensis* tea’s seed oil	HAE	hexane and DES (1:2 choline chloride- glycerol)	UHPLC-Q-TOF-MS/MS, Poroshell 120 EC-C18 column (100 mm × 2.1 mm, 2.7 μm), 30 °C, 0.1% acetic acid in water and ACN, 17 min	n-d	luteolin, vitexin, and hesperidin
		T: 50 °C				
		t: 60 min (water bath), vortex for 15 min				

Falcinelli et al. (2020)^84^	oranges and lemon seeds	UAE	methanol	HPLC-DAD Kinetex C18 column (250 mm × 4.6 mm, 5 μm), 25 °C, ACN and water/acetic acid (99:1 v/v), 260 and 325 nm, 44 min	lemon: 1.2 mg GAE/g orange: 2.5 mg GAE/g	caffeic acid, *p*-coumaric, and ferulic acids
		T: 40 °C				
		t: 30 min				
		Po: n-d				
		10 mL/g				

Santana et al. (2019)^85^	guarana seeds	PLE assisted with enzymes	water pH 5.0 with citrate buffer (pH 4.8) enzyme: 1 CMCU/mL (cellulase) and 1 GAU/mL (pectinase)	HPLC-DAD, C-18 Acclaim (4.6 mm × 150 mm, 3 μm), 30 °C, water and methanol, 210, 260, 280, and 330 nm, 60 min	56 mg GAE/g	catechin, epicatechin and epicatechin gallate
		T: 50 °C				
		t: 60 min				
		P: 10 MPa				
		100 mL/g				

Okur et al. (2021)^41^	spent coffee grounds	PLE (25 °C, 15 min, P: 500 MPa)	methanol 80%	HPLC-DAD, Eclipse XDB-C18 column (250 mm × 4.60 mm, 5 μm), 30 °C, 3% acetic acid in water and methanol, 278 nm, 81 min	950 mg GAE/g	chlorogenic acid and caffeic acid
		UAE 25 °C				
		15 min				
		Po: 400 W				
		A: 60%				
		F: 24 kHz)				

Guzmán-Lorite et al. (2022)^43^	pomegranate seeds	PLE	ethanol and bicarbonate buffer (pH 11.0)	HPLC-MS, ES-C18 column (100 mm × 2.1 mm) and a column (5 mm × 2.1 mm, 2.7 μm), 25 °C, 0.3% acetic acid in water and ACN, 43 min	14.2 mg GAE/g	caffeic acid, *p*-coumaric, and ferulic acids
		T: 170 °C				
		t: 36 min				
		P: 10.3 MPa				

a PLE: pressurized liquid extraction; T: temperature; t: extraction time; P: pressure; HPLC: high-performance liquid chromatography; ESI/MS: electrospray ionization tandem mass spectrometry; HAE: homogenate-assisted extraction; MAE: microwave-assisted Extraction; UAE: ultrasound-assisted extraction; Po: Power; A: amplitude; F: frequency; ECD: electrochemical detection; QTOF: quadrupole-time-of-flight-mass spectrometry; RT: room temperature; DAD: diode array detector; UHPLC: Ultra-High Performance Liquid Chromatography; ACN: acetonitrile; TPC: total phenolic content; TFC: total flavonoid content; GAE: gallic acid equivalent; n-d: nondetermined, CMCU: carboxymethylcellulose activity, GAU: galacturonic acid activity.

## Extraction Time

Extraction time is another critical variable and depends on the study’s aim. Strieder Strieder et al. (2024)^44^ used a long PLE-SPE time with a more analytical objective, aiming to fully extract the coffee husk’s target compounds, chlorogenic acid, and caffeine. Then, they proposed a fractionation, removing caffeine by SPE. On the other hand, most studies choose the time aiming at the efficiency of the process through the extraction kinetic curve, finishing the extraction at the end of the falling extraction rate, and the start of the diffusional controlled phase.^45^ However, in general, intensified techniques such as MAE, UAE, PLE, and PEF, compared to conventional ones such as Soxhlet and maceration, have reduced the extraction time by increasing extraction efficiency. Since extraction intensifying techniques have been employed, short times ranging from 2 min to about 40 min have been sufficient to obtain high extraction yields (Table 1–4).

## Phenolic Compounds Analyses

Despite the relevance of analyzing the extract by chromatographic techniques, most studies presented in Tables 1–4 just qualitatively analyzed the extract obtained in the best extraction condition. Researchers can associate the extract’s bioactivity by analyzing the highest-global yield extract compounds. However, quantifying compounds using standard curves can add more information about the number and quantity of compounds related to the antioxidant, antimicrobial, and antineurodegenerative effects often evaluated by researchers. Furthermore, some extraction conditions may favor the extraction of specific groups of phenolic compounds, which are usually nonassociated with the solids yield. Heravi et al. (2022),^75^ for instance, observed global extraction efficiencies from a grape pomace of 20.33%, 10.77%, and 15.49% w/w by employing ethanol, ethanol–water 50:50 v/v, and water, respectively. However, the HPLC-UV results showed the opposite results. The final concentrations of phenolic compounds in ethanol, water–ethanol, and water extracts were 650, 860, and 876 ppm, respectively. Once plant matrices present many other soluble compounds in the solvents, this example also demonstrated that the quantification of extracted solids can not be used to illustrate extraction efficiency when the objective is to obtain phenolic compounds. Maravić et al. (2022)^22^ also observed that individual phenolic profiles of sugar beet leaf extracts depended on the applied extraction technique (solid/liquid extraction, UAE, MW, PLE, or SWE). Still, vitexin was the most abundant phenolic compound determined in all extracts. Therefore, identifying and quantifying the extracted compounds obtained under different conditions is interesting.

On the other hand, by knowing the wavelength at which the target compound absorbs energy, monitoring the extraction during the development of the extractive methods using spectrophotometric techniques with UV–vis detectors can be sustainably advantageous. In this sense, Strieder et al. (2024)^86^ and Souza et al. (2021)^87^ have proposed inline detection analysis of extracts through an integrated PLE–UV–vis system. This inline procedure is a more economical and environmentally advantageous option concerning HPLC analyses, since the methodology does not employ additional solvents for analysis; the same solvent used in the extraction passes through the detector, generating a signal. In this sense, an extraction curve indicating the moment the compound is no longer observed in the extract is achieved; i.e., the extract no longer generates a signal at that particular wavelength. Strieder et al. (2024),^86^ extracting caffeine and chlorogenic acid from a coffee residue, observed similar kinetic profiles by inline analysis using a UV–vis detector and offline analysis employing a UPLC–PDA system and the standards of the compounds. Furthermore, in the offline analysis, it was observed that more phenolic compounds absorbed energy at the chosen wavelengths (270 nm for caffeine and 350 nm for chlorogenic acid), but even so, the inline curve represented the extraction of caffeine and chlorogenic acid well. Thus, in this case, the UV–vis detector replaced conventional UPLC-PDA analyses that require acidified organic solvents (methanol and acetonitrile), higher energy expenditure, and more expensive equipment than simple UV–vis detectors. However, this type of analysis would be used to optimize the extraction process, thus not precluding a more complete analysis of the extract by HPLC-mass spectrometry.

Most studies presented in Tables 1–4 employ high-performance or ultrahigh-performance liquid chromatography (HPLC or UPLC) associated with UV–visible detector (UV–vis), diode array detector (DAD or PDA), and mass spectrometry (ESI-QTOF-MS) detectors using acidified water and acetonitrile/methanol as mobile phases. However, further tendency suggest the replacement of methanol and acetonitrile for ethanol, trying to provide a greener analysis method.^13,21^ Most compounds were separated on C18 columns at approximately 30 °C and detected at 280 nm. Analysis time varies from 2 to 90 min depending on the column dimension, flow rate, temperature, mobile phase, and the samples’ compounds. Radojković et al. (2018)^52^ used the longest chromatographic run time of 90 min to analyze the phenolic compounds from Serbian mulberry (*Morus nigra*) leaves. They employed a C18 column (25 cm × 4.6 mm, 5 μm) at 25 °C with ethanol and water with 0.1% formic acid at 1.0 mL min^–1^. On the other hand, the lowest chromatographic run was proposed by de Souza Mesquita et al. (2023)^21^ using an isocratic chromatographic method with acidified water (0.25 mol L^–1^ citric acid) and ethanol at a proportion of 85:15% and at 1.5 mL min^–1^ for 2 min in a C18 column (100 mm × 4.6 mm, 2.6 μm) at 50 °C to analyze anthocyanins. In this sense, the most significant differences between the two analyses are in the characteristics of the column since the first is much longer than the second and has a larger particle diameter. Greater length requires longer analysis time, as it requires a more mobile phase to pass through the column. Moreover, smaller particle diameters, presenting higher superficial areas, favor adsorption and separation of compounds. Furthermore, the second study uses a higher temperature, accelerating mass transfer and shortening the analysis method. Finally, reviewing Tables 1–4, the chromatographic techniques vary greatly depending on the sample, column characteristics, and analysis method. However, future trends point to reducing organic solvents, such as acetonitrile and methanol, leaving a gap for new studies analyzing phenolic compounds.

## Future Perspectives

A network was built based on the studies evaluated in this review, demonstrating that the main phenolic compounds found in these residues are gallic acid, methyl gallate, *p*-coumaric acid, chlorogenic acid, protocatechuic acid, kaempferol, taxifolin, vanillic acid, ferulic acid, epicatechin, caffeic acid, rutin, quercetin, and catechin. Furthermore, verifying the predominance of some metabolites in certain groups of residues was possible. However, further extraction studies are expected to focus on identifying the compounds extracted from the materials for a more robust analysis.

Many studies about extracting phenolic compounds from plant leaves and stalks, peels and husks, pulps and pomaces, and seeds were found in the search carried out in Scopus (Figure 1). However, the number of results was significantly reduced when we limited the search by looking for studies that also carried out chromatography analyses to identify the extracted compounds. This behavior was mainly observed in studies that evaluated phenolic compounds in the seeds. Most of them report the results as TPC, and many also carry out in vitro determinations of antioxidant activity. However, they do not identify which compounds may promote the observed activities. In this sense, future studies should focus on chromatographic analysis to determine the extracted compounds.

Regarding technology and extraction conditions, including temperature, time, and solvent, extraction methods have no clear differentiation targeting specific compounds from different plant groups. PLE, SWE, UAE, and MAE are the emerging technologies studied for extracting phenolic compounds from plant waste. These technologies and their variables were discussed during the review, showing that comparison studies assessing the efficiency of procedures are challenging but essential to comprehending the effects of each technology on these compounds. These studies should evaluate efficacy not solely based on TPC yield but also target compounds by chromatographic techniques. In this sense, it would be easier to identify the effects of the extraction conditions on phenolic compounds. Furthermore, using software as a tool for solvent selection targeting specific compounds is a growing trend. This review contributed to that by identifying the key compounds present in each group of plant residues. The chromatographic analyses used to identify and quantify the compounds vary according to sample, column characteristics, and analysis conditions. However, further sustainability tendency points to using less acetone and methanol by replacing these solvents, reducing the analysis time, or employing inline detections using UV–vis detectors.

## Supporting Information Available

Table with all articles evaluated to construct Figure 2; leaves and stalk by SCOPUS 2013–2023; peel and husks by SCOPUS 2013–2023; pulp and pomace by SCOPUS 2013–2023; seeds by SCOPUS 2013–2023 (XLSX)
